# Maternal care in Acanthosomatinae (Insecta: Heteroptera: Acanthosomatidae)—correlated evolution with morphological change

**DOI:** 10.1186/s12862-015-0537-4

**Published:** 2015-11-19

**Authors:** Jing-Fu Tsai, Shin-ichi Kudo, Kazunori Yoshizawa

**Affiliations:** Systematic Entomology, School of Agriculture, Hokkaido University, Sapporo, 060-8589 Japan; Department of Biology, Naruto University of Education, Naruto, Tokushima 772-8502 Japan; Department of Biology, National Museum of Natural Science, Taichung, 40427 Taiwan

**Keywords:** Molecular phylogeny, Acanthosomatinae, Maternal care, Egg guarding, Egg smearing, Pendergrast’s organ, Correlated evolution

## Abstract

**Background:**

Maternal care (egg-nymph guarding behavior) has been recorded in some genera of Acanthosomatidae. However, the origin of the maternal care in the family has remained unclear due to the lack of phylogenetic hypotheses. Another reproductive mode is found in non-caring species whose females smear their eggs before leaving them. They possess pairs of complex organs on the abdominal venter called Pendergrast’s organ (PO) and spread the secretion of this organ onto each egg with their hind legs, which is supposed to provide a protective function against enemies. Some authors claim that the absence of PO may be associated with the presence of maternal care. No study, however, has tested this hypothesis of a correlated evolution between the two traits.

**Results:**

We reconstructed the molecular phylogeny of the subfamily Acanthosomatinae using five genetic markers sequenced from 44 species and one subspecies with and without maternal care. Eight additional species from the other two acanthosomatid subfamilies were included as outgroups. Our results indicated that maternal care has evolved independently at least three times within Acanthosomatinae and once in the outgroup species. Statistical tests for correlated evolution showed that the presence of maternal care is significantly correlated with the secondary loss or reduction of PO. Ancestral state reconstruction for the node of *Acanthosoma denticaudum* (a non-caring species in which egg smearing with developed POs occurs) and *A. firmatum* (a caring species with reduced POs) suggested egg smearing was still present in their most recent common ancestor and that maternal care in *A. firmatum* has evolved relatively recently.

**Conclusions:**

We showed that maternal care is an apomorphic trait that has arisen multiple times from the presence of PO within the subfamily Acanthosomatinae. The acquisition of maternal care is correlated with the reduction or loss of PO, which suggests an evolutionary trade-off between the two traits resulting from physiological costs. This prediction also implies that presence of maternal care can be highly expected for those groups lacking behavioral data, which invariably also lack the organ. No secondary loss of maternal care was detected in the present tree. We suggest that the loss of maternal care may be suppressed due to the vulnerability of the PO-free condition, which thus maintains maternal care.

**Electronic supplementary material:**

The online version of this article (doi:10.1186/s12862-015-0537-4) contains supplementary material, which is available to authorized users.

## Background

Parental care in insects has been the focus of several studies that examined its adaptive functions with regard to both parents and offspring [1]. Such studies may clarify selection regimes that maintain parental care under current ecological conditions and shape co-adaptive behavioral interactions between parents and offspring. However, the selection acting on current populations may not be the same as that in the origin. The phylogeny-based comparative analysis is a powerful tool for testing or generating the hypotheses about the historical developments of traits. However, relatively few attempts have been made to apply this analysis to the evolution of parental care in insects [2, 3].

Evolutionary transitions and the lability of uni- or biparental care have attracted the interest of evolutionary biologists [4–6]. Complex parental care, with integrated morphological (e.g., placenta) and behavioral components, may have a low likelihood of loss. For example, viviparity (a common form of maternal care associated with internal fertilization) has evolved many times, but it has never been lost in ray-finned fish [7]. Parental food provisioning which has been elaborated through co-evolution between parents and their offspring confers resistance to loss [8]. In contrast, simple attendance and guarding of offspring might be more easily lost in low-risk environments than complex parental care [6]. Tallamy and Schaefer [9] suggested that parental care is a plesiomorphic relic in Hemiptera, which has repeatedly been lost due to the high cost of caring.

Post-ovipositional parental care has been recorded in at least 64 genera representing 14 families of four infraorders of heteropteran insects [9–16]. Most of them exhibit maternal care, whereas exclusive paternal care is restricted to four families only with reports of dozens of genera: Belostomatidae, Coreidae, Reduviidae, and Pentatomidae [15, 17, 18]. Approximately 70 % of the species in which maternal care has been documented belongs to the superfamily Pentatomoidea. They have developed diversified strategies of maternal investments, such as physical protection against predators with defensive movements in many taxa (e.g., [13, 19, 20]), brood caring combined with nymphal phoresy in the Phloeidae and Tessaratomidae (e.g., [11, 13, 21]), joint guarding in some Acanthosomatidae (e.g., [22, 23]), or a series of complex cares including egg-translocation, trophic egg production, hatching assistance, progressive provisioning and joint breeding in cydnoid families (e.g., [24–35]).

The family Acanthosomatidae is one of the best known members of Pentatomoidea, in which the females of several species display a simple form of parental care, egg-nymph guarding, with effective resource allocation among eggs [10, 23, 36–43]; for an extensive review see [20]. Most of them are oligo-or polyphagous, arboreal herbivores that feeding on the developing fruits of some conifers and many flowering plants (e.g., [20, 44]). The family currently contains about approximately 285 described species in 56 genera in three subfamilies, namely Acanthosomatinae, Blaudusinae and Ditomotarsinae [45–53]. Considering that its sister group, Lestoniidae, is an endemic Australian family, and that the majority (approximately 80 %) of acanthosomatid genera is distributed in the fragmented landmasses of Gondwana, the family is very likely of Gondwanan origin. However, the greatest species diversity (nearly 80 % of the total number of species) is found in the 14 genera of the subfamily Acanthosomatinae, with a high species richness in East, South, and Southeast Asia.

Many acanthosomatine species exhibit maternal care. For example, members of the genera *Elasmucha* and *Sastragala* attend their eggs and nymphs until the 2nd to 5th instar [10]. Several studies have identified the strategy’s defensive function, the agents of offspring mortality and have quantified its benefits in terms of offspring survival under field conditions [39, 41, 54–58]. In contrast, the females of other species of the subfamily do not show post-ovipositional care, and instead, smear the eggs one after another with secretion from Pendergrast’s organ using the hind legs before leaving the clutch [59–61], see also Additional files 1 and 2. The Pendergrast’s organ (PO) is a pair of disc-like, depressed, setose areas located sublaterally on the female abdominal sternites V–VII, VI–VII or VII, having highly modified cuticle (ductules, pores and setae) with numerous underlying and closely arranged secretory cells in the epidermal layer [61, 62]. Although no direct experimental evidence has been published thus far, the organ’s secretion supposedly functions as a repellent against the predators and parasitoids [61, 63, 64], which can be considered as a form of maternal care if the substances could effectively enhance egg survival.

The monophyly of Acanthosomatidae and its sister relationship with the Lestoniidae are apparently supported by molecular and morphological data [65, 66]. However, no hypothesis for the phylogenetic relationships within the family has been proposed so far, rendering it difficult to understand the origin and evolution of maternal care. In this study, we focused on the relationships of the subfamily Acanthosomatinae, the taxon containing the largest number of species. We estimated the molecular phylogeny among the major groups of Acanthosomatinae with and without maternal care and incorporated several outgroup representatives of the other two subfamilies using three mitochondrial and two nuclear genes. We evaluated whether the maternal care has a single origin or has evolved independently several times within Acanthosomatidae. We further examined whether the maternal care is associated with morphological changes, i.e., the loss of PO, since some authors have proposed a trend that species showing maternal care lack this organ [60, 61].

## Results

### Phylogenetic analyses and the clades recovered

**Fig. 1:**
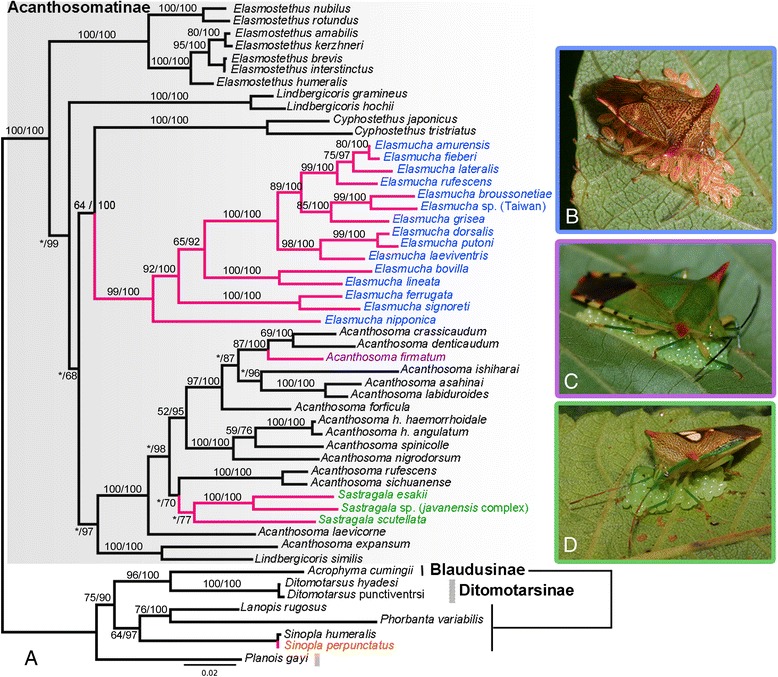
**a**. Phylogenetic tree resulting from Bayesian method based on five gene regions (mitochondrial *12S*, *16S rRNA*, *COI*, nuclear *18S rRNA*, *Histone 3*). **b**–**d**, Three acanthosomatine females guarding their egg batches or nymph clusters. **a**, topologies resulting from Bayesian and ML methods are identical. Numbers associated with each branch indicate ML bootstrap/Bayesian posterior probabilities, values below 50 % marked with asterisk; clades in red indicate presence of maternal care. **b**, female of *Elasmucha bovilla* guarding her first instar nymphs. **c**, egg-guarding female of *Acanthosoma firmatum*. **d**, egg-guarding female of *Sastragala esakii*

The phylogenetic trees resulting from Maximum Likelihood (ML) and Bayesian analyses were nearly identical and were well resolved except for some basal branches (Fig. 1a). The monophyly of the genera *Cyphostethus*, *Elasmostethus* and *Elasmucha* were strongly supported (100 % bootstrap [BP] and posterior probability [PP]) in all analyses. The monophyly of *Sastragala* was estimated as most likely but this was not robust (77 % PP). The monophyly of *Acanthosoma* and *Lindbergicoris* was not recovered due to a robust sister relationship between *A. expansum* and *L. similis* (100 % BS and PP). The latter two species never formed a monophyletic group with the rest of *Acanthosoma* and *Lindbergicoris*, respectively. The monophyly of *Lindbergicoris* containing *L. gramineus* and *L. hochii* was recovered (100 % BS and PP). The placements of *A. laevicorne* and a clade of the sister species *A. rufescens + A. sichuanense* are still inconclusive; the relationships either with the other members of *Acanthosoma* or *Sastragala* were weakly supported. A clade consisting of *Acanthosoma* (excluding *A. expansum*) and *Sastragala* is strongly supported (100 % BS and PP).

The sister relationship between *Cyphostethus* and *Elasmucha* was moderately to strongly supported (64 BS and 100 % PP). The internal branches of *Elasmucha* were well resolved and well supported. Several clades can be recognized in this species-rich genus. The sister relationship between *Elasmostethus* and the other acanthosomatine genera is supported by the Bayesian method (99 %) but is weakly supported by ML bootstrapping (27 %). The internal relationship of *Elasmostethus* was fully resolved with strong support.

### Evolution of maternal care and morphological correlation

From the resulting tree, three independent origins of maternal care in the subfamily Acanthosomatinae were estimated as most parsimonious: one in the common ancestor of *Elasmucha*, one in the common ancestor of *Sastragala*, and one in the species *Acanthosoma firmatum* (Fig. 1). Shimodaira-Hasegawa and Kishino-Hasegawa tests (constraining all species with maternal care in Acanthosomatinae as a monophyletic group) rejected the monophylgetic origin of the maternal care in the subfamily (Fig. 1a) (*p* < 0.001 in both the SH and KH tests). At present *A. firmatum* (Walker), frequently referred to in the literature as *A. giganteum* Matsumura [53], is the only species exhibiting maternal care in the genus *Acanthosoma* [67]. Similarly, *Sinopla perpunctatus* in the subfamily Blaudusinae is also the single representative in its genus in which maternal care is known [68].

**Fig. 2:**
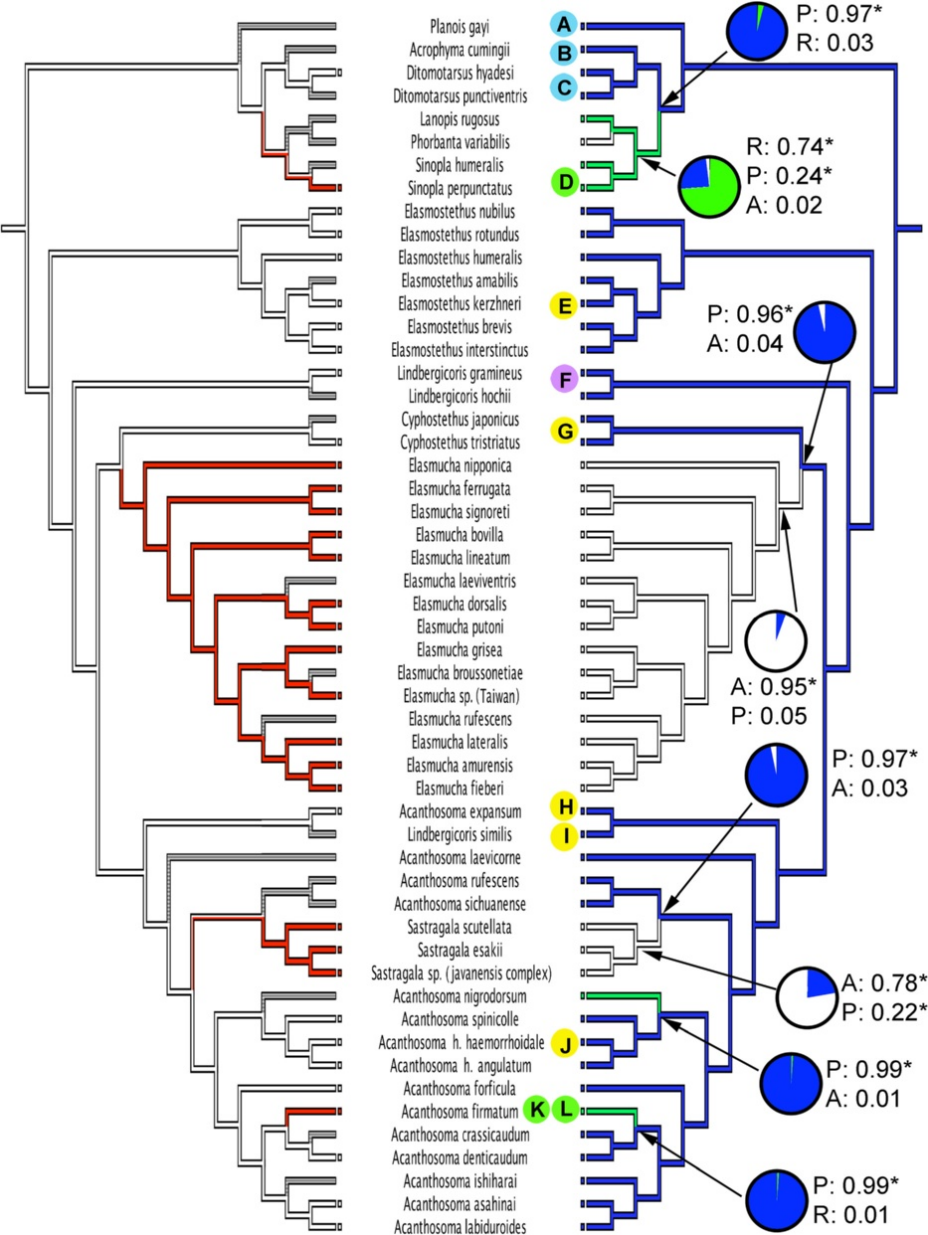
Character evolution of maternal care (*left*) and Pendergrast’s organ (*right*) using parsimony based on the topology as shown in Fig. 1a. Left cladogram showing four steps under parsimony reconstruction (4 gains): red clades indicate presence of egg-nymph guarding (maternal care), white indicate absence, clades with a mixture of two colors indicate equivocal, clade in gray indicates unknown of reproductive behavior. Right cladogram showing 6 steps under parsimony reconstruction (three losses and three reductions): blue clades indicate presence of Pendergrast’s organ (PO), green indicates reduced PO, white indicates absence of PO, pie charts and values show likelihood reconstruction of nodes of interest, states judged best estimate under the threshold (2) are marked with an asterisk, letters indicate representative forms of PO shown in Fig. 3, letters with same color background have the same pattern of PO

To understand the correlation between the behavioral and morphological evolution, the character states of PO (which is related to egg smearing) was mapped on the best Bayesian tree (Fig. 2). The correlated evolution analyses [69] demonstrated that the absence of the organ is significantly correlated with the presence of maternal care (actual changes: *p* = 0.0357, MINSTATE reconstructed-changes: *p* = 0.022, MAXSTATE reconstructed-changes: *p* = 0.0354). In addition, the difference in likelihood between the independent and dependent model was shown by Pagel’s test as significantly greater (difference in likelihood = 12.854, *p* < 0.001). Two characters under any effect factor significantly fit the dependent (= correlated) model better; the independent model was therefore rejected.

**Fig. 3:**
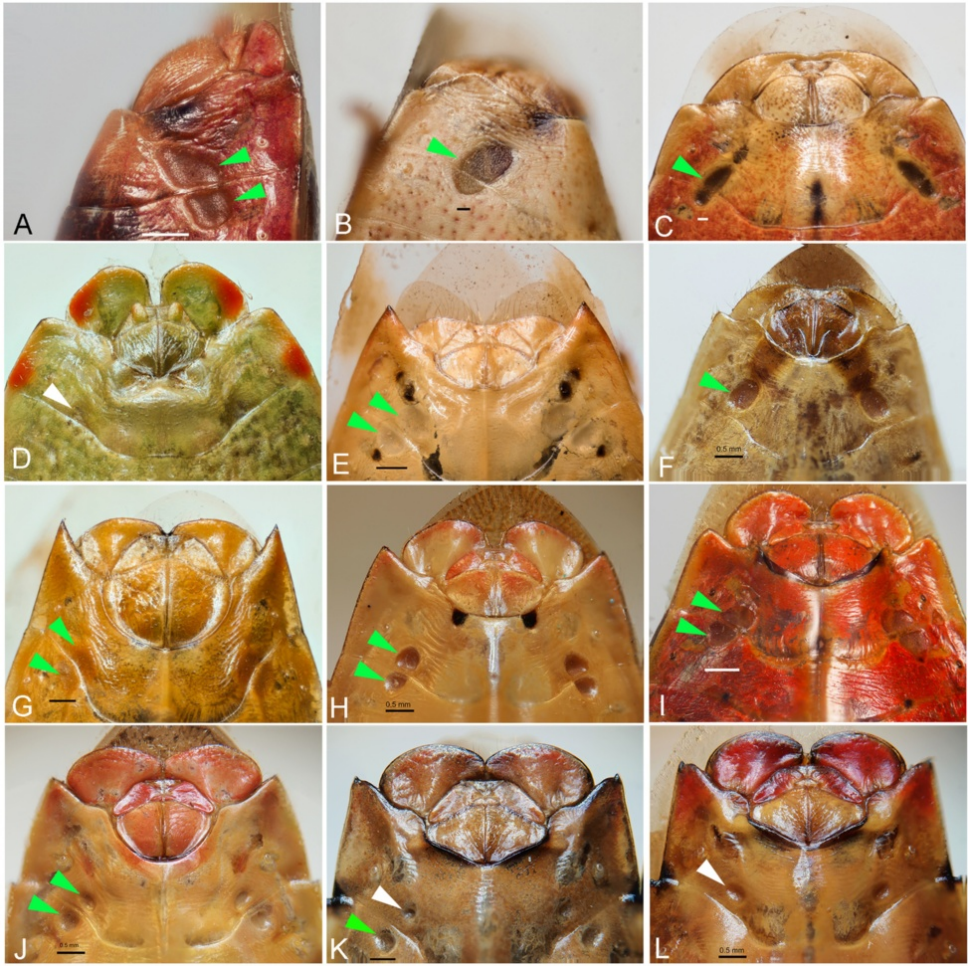
Various forms of Pendergrast’s organ (PO) located on the abdominal venter of females of 11 species of Acanthosomatidae. Green arrow indicates presence of a well-developed PO on abdominal sternites VI and VII, white arrow indicates reduced PO. Scales in 0.5 mm, **a**–**b**: lateral view, **c**–**l**: ventral view. **a**, *Planois gayi*; **b**, *Acrophyma cumingii*; **c**, *Ditomotarsus punctiventris*; **d**, *Sinopla perpunctatus*; **e**, *Elasmostethus kerzhneri*; **f**, *Lindbergicoris gramineus*; **g**, *Cyphostethus triastriatus*; **h**, *Acanthosoma expansum*; **i**, *L. hastatus*; **j**, *Acanthosoma haemorroidale angulatum*; **k**–**l**, *Acanthosoma firmatum*. Species generally display two pairs of elliptic POs on sternites VI and VII (**e**, **g**, **h**, **i**, **j**), which are occasionally fused into a single large area (**b**, **c**), or approach each other closely (**a**). In other species a single pair of large, rounded PO is present on sternite VII (**f**). Individuals of *A. firmatum* exhibits a gradual reduction of PO: either reduced in size on sternite VII (**k**) or lost one pair on sternite VI (**l**). *S. perpunctatus* only has a vestige of PO on sternite VII (**d**). We provided photo of a closely species, *L. hastatus* instead of *L. similis* due to unavailability of females of the latter species

Five patterns of PO are shown in Fig. 3, which correspond to the character evolution shown in Fig. 2. In general, species display two pairs of elliptic POs on sternite VI and VII (Fig. 3e, g, h, i), or fused into one large area (Fig. 3b, c), or closely joint (Fig. 3a). In the other examples, species exhibit a pair of large POs on sternite VII (Fig. 3f). Two exceptions are found in *A. firmatum* and *S. perpunctatus*, in which POs remain but they still exhibit maternal care. However, they frequently have a reduced pair on abdominal sternite VII and have lost one pair on abdominal sternite VI (Fig. 3d, k–l).

**Fig. 4:**
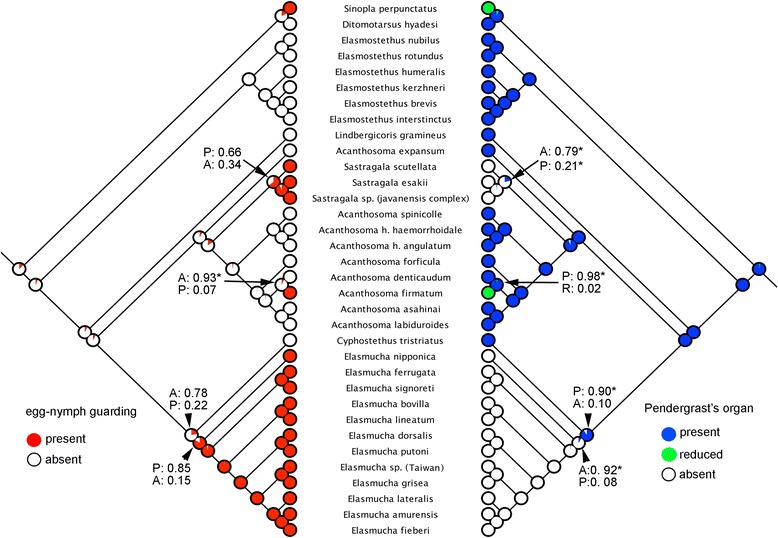
Likelihood reconstruction of ancestral state of egg-guarding behaviour (*left*) and Pendergrast’s organ (*right*) in 34 terminal taxa without missing data. Values on the nodes of interest were shown, and indicate proportional likelihood. The states judged best estimate under the threshold (2) are marked with an asterisk

The reconstruction of the ancestral state of Pendergrast’s organ (PO) performed by likelihood calculation is given in Fig. 2. The best estimates of the proportional likelihoods for nodes containing maternal care species are as follows: (1) the likelihood for the node of *Acanthosoma firmatum* and *A. denticaudum* + *A. crasssicaudum* is 0.99 (present) and 0.01 (reduced); (2) the likelihood for the node of *Sastragala* is 0.22 (present) and 0.78 (absent); (3) the likelihood for the node of *Elasmucha* is 0.05 (present) and 0.95 (absent); (4) and for the node of *Sinopla perpunctatus* + *S. humeralis* and *Lanopis rugosus* + *Phorbanta variablis*, the likelihood is 0.24 (present), 0.74 (reduced), and 0.02 (absent). The likelihood reconstruction of the ancestral state of maternal care and PO was also estimated using the trimmed tree (a total of 34 terminal taxa without missing data) (Fig. 4). The proportional likelihoods for the node for maternal care and its corresponding node for the presence of PO within the Acanthosomatinae are as follows: (1) node for the presence of maternal care for *Sastragala* is 0.66 and its absence is 0.34; the node for absence of PO is 0.79, for its presence, 0.21; (2) for *A. denticaudum* and *A. firmatum*, the node for the presence of maternal care is 0.07, and its absence is 0.93; the node for the presence of PO is 0.98, and for reduction, 0.02; (3) for *Elasmucha*, the node for the presence of maternal care is 0.85, and for its absence, 0.15; the node for the absence of PO is 0.92 and for its presence, 0.08.

## Discussion

### Multiple origins of maternal care in the acanthosomatidae

Statistical tests rejected the possibility of a single origin for maternal egg-nymph guarding (maternal care) behavior in the Acanthosomatidae, but indicated that it has independently evolved at least three times in the subfamily Acanthosomatinae (*Elasmucha*, *Sastragala*, and *Acanthosoma firmatum*) and once in Blaudusinae (*Sinopla perpunctatus*). The multiple and independent origins of maternal care in distantly related lineages suggest that this guarding behavior did not appear as a result of phylogenetic conservatism, but it was probably driven by complex selective factors (e.g., [70]; see below).

Because Pendergrast’s organ is homologous with the disc-like organ of Lestoniidae, the sister group of Acanthosomatidae, it therefore belongs to the ground plan of the clade Lestoniidae + Acanthosomatidae. It is presumably a ground plan character for Acanthosomatidae too [61, 65]; consequently, its loss is derived. Ancestral state reconstruction suggests that maternal care has mostly likely acquired in the presence of Pendergrast’s organ (Fig. 2). Moreover, no secondary loss was detected once maternal care had arisen. As maternal care seems to be resistant to loss at least in acanthosomatid bugs, and additionally it is apparently a recent acquisition in certain acanthosomatid lineages (see discussion below), our results do not support the hypothesis of Tallamy and Schaefer [9] that parental care is a plesiomorphic relic character and is labile, i.e., it has repeatedly appeared and been lost in Hemiptera.

### Correlation between maternal care and secretory organ

As mentioned above, the secretory Pendergrast’s organ (PO) is a plesiomorphic feature for the Acanthosomatidae, therefore egg smearing is an ancestral behavior (relative to egg guarding) in the family. Species showing maternal care either lack or have a reduced, non-functional PO. This organ allows the female to perform egg-smearing by coating the eggs with substances that probably have a protective function [61, 63]. The correlation analyses convincingly indicated that the presence of maternal care is significantly correlated with the loss or reduction of the PO. Considering the strong correlation between a morphological and a behavioral trait, maternal care can be expected to occur in other genera of the family, such as *Agamedes*, *Bebaeus*, *Catadipson*, *Ibocoris*, *Mahea*, *Phorbanta*, *Proctophantasta* and *Uhlunga*, all of which invariably lack PO (also suggested by Fischer [61]). The negative correlation between the egg-nymph guarding and PO with egg-smearing behavior suggests an evolutionary trade-off between the two traits, resulting from the high physiological costs of producing and maintaining both behaviors under allocation of limited resources. Alternatively, it could be explained by a relaxed selection against redundant traits. Once alternative strategy has evolved, selective pressure for maintaining the other strategy should be relaxed. Reduction of one of the redundant traits should also be selectively advantageous for efficient allocation of resources.

Likelihood reconstruction of the ancestral state of Pendergrast’s organ for the ancestral node of *Acanthosoma firmatum* (with reduced POs), *A. denticaudum* and *A. crassicaudum* (the latter two sibling species with well-developed POs) showed a significantly high proportional likelihood for the organ’s presence (0.99) (Fig. 2). The trimmed tree with all available behavioral data also showed a concordant pattern between the absence of maternal care (0.93) and the presence of PO (0.98) at their ancestral node (Fig. 4). This finding indicates that the egg-smearing behavior was still present in the common ancestor of *A. firmatum* and *A. denticaudum*. Consequently, the maternal care in *A. firmatum* mostly likely has developed relatively recently and was followed by a subsequent reduction of the PO. The case of *Acanthosoma firmatum*, where females display maternal care and PO has been reduced to various degrees, might support a predicted phenotypic trade-off between different degrees of maternal care and reduction of Pendergrast’s organ among and/or within populations.

A similar scenario presumably occurred in the common ancestor of *Lanopis* (with two pairs of reduced POs)*, Phorbanta* (without a PO) and *Sinopla* (with either a reduced or a lost PO). The ancestral state reconstruction showed a significantly high proportional likelihood of reduced PO (Fig. 2, 0.74). The prediction suggests that the acquisition of maternal care precedes reduction and the loss of PO.

### How did the maternal care evolve in acanthosomatids?

The evolution of paternal care in hemipteran lineages has been driven by a complex of factors. It probably originated as a response to pressure from predators and parasitoids [43, 71–76], to prevent eggs from desiccation [77, 78], to develop a more elaborate manipulation of tradeoffs between air exchange and desiccation in water bugs [79–82], or to represent an adaptation to unstable or ephemeral food resources in cydnid families [25, 27, 32, 83, 84]. In treehoppers, maternal care is associated with changes from a solitary to gregarious life history in connection with the acquisition of ant mutualism [2, 74].

In several species of *Elasmucha* (Acanthosomatinae), field experiments have demonstrated that eggs and hatched nymphs are subject to intense predation; females effectively guard them against arthropod predators [39, 41, 54–57, 85] but not against parasitoids of the nymphs [58]. These suggest that the high predation pressure is a primary factor for the acquisition of maternal care in these insects.

In addition to the selection pressure derived from the change in environmental conditions, an ancestral reproductive mode, i.e., deserting eggs after smearing, may also be associated with the emergence of maternal care. Both egg smearing and guarding behaviors share an intimate contact between the female and her eggs at the oviposition site. The smearing process itself forces the female to invest extra energy and time on each egg until forming an egg-clutch (JFT, unpublished observation). Such a prolonged stay at the oviposition site could be an exapted condition that promotes the development of maternal care.

### Why is maternal care not lost once it has evolved in acanthosomatines?

As our results show, maternal care has arisen from a condition with the presence of Pendergrast’s organ (Fig. 2) and a strong negative correlation was found between the two traits. We presumed that both egg-smearing and egg-guarding have their own selective advantages in acanthosomatid bugs, however, once maternal care has been acquired, the Pendergrast’s organ and therefore the egg-smearing will be reduced or lost because of a trade-off relationship resulting of high physiological costs of both traits. The loss of egg-nymph guarding in Acanthosomatinae may also likely be inhibited due to a resulting loss of PO, a presumably vulnerable condition which lacks any protective substances as well as female attendance. This could be why no secondary loss of the guarding behavior is observed. Even when selection favors the reduction and loss of maternal guarding as a result of a decrease in predation pressure, re-evolution of the morphologically complex PO may not be possible. Consequently, even if resources were constantly available, a potential non-caring reproductive strategy (i.e., depositing many egg batches without any protection) could not offset offspring mortalities and thus could not enjoy greater reproductive success than that from a caring strategy even under conditions of moderate predation.

In contrast, the apomorphic trait of maternal care has been lost at least once in the treehopper subfamily Membracinae due to its drastic life history specialization with the acquisition of ant mutualism [2]. Ant mutualism is apparently an alternative life history, which increases the survival of offspring more effectively than maternal guarding and consequently resulted in the secondary loss of the latter. However, such a strategy has not been observed in acanthosomatid bugs.

## Conclusions

The family Acanthosomatidae is one of the best known members of Pentatomoidea, in which females of several taxa display egg-nymph guarding behavior. However, the origin and evolution of maternal care remain unclear due to the lack of a phylogenetic hypothesis. In this study, we proposed a molecular phylogeny of Acanthosomatinae for the first time. Maternal care has independently evolved at least four times within the lineages of this family. Statistical analyses rejected the possibility of a single origin within Acanthosomatinae and revealed at least three independent origins among the distantly related lineages. Our results revealed that maternal care is an apomorphy (relative to egg smearing), which has arisen in the presence of secretory Pendergrast’s organ, where their common ancestor still exhibits a plesiomorphic reproductive trait, i.e., deserting the eggs after smearing. A negative evolutionary correlation suggests a trade-off between the acquisition of maternal care and the reduction or loss of the secretory organ. Alternatively, this negative correlation is possibly a consequence of relaxed selection against one of two redundant traits. The presence of maternal care is to be highly expected in other genera that also lack or have a reduced, unfunctional organ. Previous studies indicate that the evolution of maternal guarding is driven by high predation pressure. Although maternal guarding might be easily lost in a low-risk environment, it seems resistant to such lost in acanthosomatids. We found that no secondary loss of maternal care occur once it has evolved. The maintenance of maternal care in acanthosomatids are likely due to a vulnerable Pendergrast’s-organ-free condition. Our phylogenetic hypothesis provides a basis for future comparative analyses of the evolution of parental care and other reproductive traits. The multiple origins of maternal care estimated here will enable us to further test general hypotheses about the ecological and life-history conditions favoring care as well as about the evolutionary trends with other reproductive traits, such as egg size.

## Methods

### Taxon sampling

A total of 53 terminal taxa were included in the study (Additional file 3). The ingroup consisted of 44 species and one subspecies belonging to six genera of Acanthosomatinae and covered all representative members for which published data are available on maternal care. An additional eight species in six genera belonging to the other two subfamilies were included as outgroups and were used to root the phylogenetic tree.

### Molecular markers and primers

Five genes including mitochondrial protein-coding (Cytochrome Oxidase I [*COI*]), two ribosomal genes (*12S* and *16S*), nuclear protein-coding (Histone 3 [*H3*]) and ribosomal genes (*18S*) were sequenced. Primer sets for the target regions, *COI* (LCO1490-HCO2198, [86]), *12S* (12Sai-12Sbi, [92]), *16S* (16Sar-16Sbr, [87]), *H3* (HexAF-HexAR, [88]), and *18S* (NS1-NS2a, [89]) were used for amplification and sequencing.

### DNA extraction and purification, PCR amplification, and sequencing

All specimens were preserved in 99.5 % ethanol in the field, followed by long-term storage at −20 °C. The thoracic muscles and legs were digested in Proteinase K solution for 12–18 h at 56 °C in an incubator and then used for DNA extraction following the standard protocols suggested by the Qiagen DNeasy Tissue kit (Qiagen). PCR reaction cycles were performed with an initial denaturing step at 94 °C for 3 min, followed by 35 cycles of 94 °C for 30 s, 42 °C (*16S*), 45 °C (*12S*), 50 °C (*COI*, *18S*) or 54 °C (*H3*) for 30 s and 72 °C for 1 min. DNA samples were sequenced by CEQ 2000XL DNA Analysis System (Beckman Coulter, California, USA) following the manufacturer’s protocols.

### Sequence alignment and phylogenetic analyses

Alignments of Histone 3 and *COI* were straightforward and based on amino acid sequences. Mitochondrial rDNA was aligned using ClustalX 2.1 [90] with Gap:Gap-extension costs = 10:1 and 20:0.1 to recover the maximum numbers of stem regions [91]. The same software and cost-set was also applied to the alignment of 18S rDNA. The alignment was adjusted manually by eye, and ambiguously aligned regions were excluded from the analyses based on similarity criterion [92], resulting in a concatenated alignment of 2182 bp. Aligned data in nexus format are available as Additional file 4.

We performed maximum likelihood (ML) and Bayesian analyses. For ML analyses, a heuristic search with Tree Bisection and Recombination (TBR) branch swapping using a Neighbor Joining starting tree was performed by PAUP* 4.0b10 [93]. The best-fit substitution model was estimated using hierarchical likelihood ratio tests (hLRT) as implemented in jModeltest 2.1.5 [94, 95], and the GTR + I + G model was selected (unequal base frequencies: A = 0.2974, C = 0.1616, G = 0.1863, T = 0.3547; six substitution categories: A–C = 3.0456, A–G = 13.8985, A–T = 5.8488, C–G = 1.2926, C–T = 28.9260, G–T =1; gamma distributions shape parameter = 0.5330 based on four rate categories; proportion of invariant sites = 0.6260). ML-based bootstrap values were calculated using PhyML 3.0 [96] with the GTR model and estimated parameters with 1000 replications.

For Bayesian analysis, we separated the characters into nine partitions (*12S*, *16S*, *18S*, three codon positions of *H3* and *COI*, respectively). The best-fit model was estimated independently for each partition using hLRTs as implemented in MrModeltest 2.2 [97], resulting in *12S*, *16S*, the first codon of *COI* (GTR + I + R), the second codon of *COI* (F81 + G), third codon of *COI* (GTR + G), *18S* (K80 + G), the first coden of *H3* (F81), second conden of *H3* (JC), third conden of *H3* (SYM + G). Bayesian analysis was conducted using MrBayes 3.1.2 [98] with two runs of four chains each for 2,000,000 generations and tree samples every 1000 generations. The first 50 % of the trees were discarded as a burn-in, and a 50 % majority consensus tree was used to calculate posterior probabilities.

### Constraint analyses

The likelihood of competing hypotheses of maternal care evolution was tested statistically by using the constraint trees. To test whether the maternal care is of single origin, species with maternal care were constrained as monophyletic group while the rest species were collapsed to polytomies, and likelihood scores was compared with the best ML tree. All constraint ML topologies were estimated with the same substitution model and tree searching algorithm as used for the MLtree search. The non-parametric likelihood ratio test was performed by the Shimodaira-Hasegawa test (test distribution set as RELL) and Kishino-Hasegawa test (two-tailed).

### Data collection for Pendergrast’s organ and reproductive behavior

Surveys on the character distributions and states of Pendergrast’s organ in all terminal taxa, comprising 53 species, are based on our observations from the alcohol-preserved and dry museum specimens.

To determine whether females show maternal care, we collected gravid females of each species, confined them in rearing cases with host plants, and then checked for oviposition under laboratory conditions. For some species, we observed maternal care directly under field conditions. Maternal care can be easily recognized in acanthosomatids by the remarkable posture and behavior of females; caring females invariably straddle egg masses and hatchlings tightly (Fig. 1b, c, d), and when disturbed, it shows specific aggressive responses, e.g., tilting the body towards the source of disturbance [36, 37, 41, 54, 56]. On the other hand, females of asocial species always leave oviposition sites soon after depositing egg mass. Data on the reproductive behavior of eight species are referred from the literatures [20, 42, 61, 68, 99, 100, 105]; detailed information on the caring behavior in two additional species, *E. lineata* and *Sastragala* sp. was obtained from J. Horstman (*pers. comm.*). Including records from the literatures, we obtained data on reproductive behavior for 34 species (Additional file 3).

### Tracing character evolution

Behavioral data and condition of Pendergrast’s organ (PO) were listed in Additional file 3, and the corresponding of character the coding matrix refers to Additional file 5. Species lacking of behavioral data were coded as missing (19 out of 53 species). Behavior of egg-nymph guarding is treated as a binary character (present or absent). The character of PO was coded as three states: absent, present, and reduced (Additional file 5). Characters were mapped on the Bayesian tree according to the parsimony criterion produced by Mesquite 3.02 [101]; matrix refers to Additional file 6. For reconstruction of the ancestral state of PO, a likelihood criterion was performed by Mesquite 3.02. The same method was also applied to the trimmed tree (the same topology for Pagel’s correlation test) with likelihood reconstruction for maternal care and PO; matrix refers to Additional file 7. The current probability model of the Bayesian tree was used as the source of a character model for likelihood reconstruction at each node. The likelihood decision threshold is two as the default (the commonly used value proposed by Pagel [102]).

### Correlated evolution analysis

To determine whether a correlation between the presence of maternal care and absence of Pendergrast’s organ is significant, we performed two statistical methods using the concentrated-changes test [69] and likelihood-based correlation method [103]. For character coding, we modified the matrix of Additional file 5 into binary characters (Addition file 8), and treated a reduced Pendergrast’s organ as “absent” state because the scanning electron microscope observations and histological evidences suggest a loss of the secretory function in the reduced organ (JFT, unpublished observation). We removed those taxa with missing data (a total of 19 species lacking behavioral information), and maintained the shape of the Bayesian topology (Fig. 1a) as the backbone tree for the analyses of correlated evolution. In the concentrated-changes test, we performed three respective options with MacClade 4.08a [104]: actual changes, MINSTATE and MAXSTATE reconstructions for numbers of gains and losses of Pendergrast’s organ. We indicate the “0” (absent) and “equivocal” state for the choice of distinguishing branches as those having in the character traced under 0 gains and four losses over the whole cladogram for 1,000,000 simulations. To avoid an assumption of actual changes, the other two algorithms of reconstructed-changes were performed in MINSTATE and MAXSTATE and given “1” (present) as the initial state and “0” as compensation. We also performed Pagel’s correlation analysis using Mesquite 3.02 [101]. The branch length of the trimmed tree (with 34 terminal taxa) was re-estimated by the Bayesian method for the correlation test. Pagel’s test was set as “any effect” by designating Pendergrast’s organ and maternal care as either X or Y. The likelihood difference between independent and dependent (= correlated) models was estimated for 1000 simulations. Differences in the likelihood of the independent versus correlated models of evolution were estimated where *P*-values below 0.05 indicate a significant correlation between the the two traits.

### Observations of egg-smearing behavior

Egg-smearing behaviour was successfully documented in five species using a digital camera (Olympus Digital Camera TG-2): *Acanthosoma denticaudum*, *A. haemorrhoidale angulatum*, *A.labiduroides*, *Elasmostethus humeralis*, and *E. interstinctus*. Overwintering females were collected from their host plants at the campus of Hokkaido University (Sapporo), Forestry and Forest Products Research Institute (Hokkaido Research Center, Hitsujigaoka, Sapporo) and Zenibako-gawa (Otaru) from mid June to early July in 2013 and 2014. None of these are endangered or protected species and no permits were required for their study. Gravid females were reared individually in transparent Petri dishes (9 cm diameter, 3.5 cm height) supplied with shoots of Japanese rowan (*Sorbus commixta*), hornbeam (*Carpinus cordata*) or hogweed (*Heracleum dulce*) bearing fresh fruits. For determining the active oviposition period, the animals were observed every 30 min from 10:00 to 22:00. If a female was found in ovipositing posture (i.e., bending the antennae backward and against the body, standing with the hind tarsi close together under the tip of the abdomen, and exhibiting slight movements of the valvifers accompanied by stamping of the hind legs for measuring the egg-laying site), then we started recording using movie mode of an Olympus digital camera in super macro mode. Two common species, *Elasmostethus humeralis* and *Acanthosoma denticaudum*, were selected as models for demonstrating the egg-smearing behavior (see Additional files 1 and 2).

### Availability of supporting data

The data sets supporting the results of this article are available in Additional files.

## Additional files

Additional file 1:
**Format: MPEG 4.** Title: Egg-smearing behavior of *Elasmostethus humeralis*. Legend: Female of *Elasmostethus humeralis* ovipositing on a fruit of the hogweed (*Heracleum dulce*); natural speed. After the egg is laid the female spends about 1 min to spread the secretion onto each of the eggs, one after another, with its hind legs rubbing against Pendergrast’s organ (PO). First part (dorsal view): note that the diagonal movements of the left and right legs follow each other almost without interruption. Second part (close-up in lateral view): rubbing each of hind tarsi and tips of tibiae alternatively and repeatedly against PO. Third part (posterior view): initial process of egg-laying; after the egg is deposited the female immediately starts smearing it. (M4V 17537 kb) Additional file 2:
**Format: MPEG 4.** Title: Egg-smearing behavior of *Acanthosoma denticaudum*. Legend: Female of *Acanthosoma denticaudum* ovipositing on a leaf of Japanese rowan (*Sorbus commixta*). Note the short interruption (about 1 s) between the movements of both legs during smearing. (M4V 13586 kb) Additional file 3:
**Format: XLS.** Title: Species sequenced in the study, behavioral data, and condition of Pendergrast’s organ on the pregenital segments. Legend: Symbols: + (present), − (absent), R (reduced). ^1^This species is conspecific with *Acanthosoma giganteum* Matsumura, recently synonymized by Tsai & Rédei [53]; the junior synonymous name, *A. giganteum*, is frequently cited, and its mitochondrial gene of cytochrome oxidae subunit I (*mtDNA-COI*) is also available from GenBank (AB368853). ^2^An undescribed species closely related to *S. javanensis* Distant. Collection abbreviation: JFT– Jing-Fu Tsai’s coll. deposited in systematic laboratory of Hokkaido University, Sapporo, Japan; NKU– Department of Zoology and Developmental Biology, Nankai University, Tianjin, China. LC099108–LC099371: Genbank accession numbers. (XLS 56 kb) Additional file 4:
**Format: nexus.** Title: Data matrix of aligned sequences for Bayesian analysis. (NEX 130 kb) Additional file 5:
**Format: XLS.** Title: Data matrix for character evolution. Legend: Data matrix for character evolution. Character 1, egg-nymph guarding behaviour (maternal care) [0 = absent, 1 = present, ? = missing data]. Character 2, Pendergrast’s organ [0 = absent, 1 = present, 2 = reduced]. (XLSX 10 kb) Additional file 6:
**Format: nexus.** Title: Data matrix for parsimonious reconstruction of the two traits (maternal care and Pendegrast’s organ) on 53 species, including species with missing data. (NEX 20 kb) Additional file 7:
**Format: nexus.** Title: Data matrix for likelihood reconstruction of ancestral state of the two traits (maternal care and Pendegrast’s organ) on 34 species with confirmed behavior data. (NEX 16 kb) Additional file 8:
**Format: XLS.** Title: Data matrix for correlation analysis. Legend: Data matrix for correlation analysis between maternal care and Pendergrast’s organ. Character 1, egg-nymph guarding behaviour (maternal care) [0 = absent, 1 = present]. Character two, Pendergrast’s organ [0 = absent or reduced, see explanation in Methods, 1 = present]. (XLS 31 kb)
